# Analysis of pit latrine microbiota reveals depth-related variation in composition, and key parameters and taxa associated with latrine fill-up rate

**DOI:** 10.3389/fmicb.2022.960747

**Published:** 2022-09-23

**Authors:** Umer Zeeshan Ijaz, Ozan Gundogdu, Ciara Keating, Miriam van Eekert, Walter Gibson, Julian Parkhill, Faraji Abilahi, Benard Liseki, Viet-Anh Nguyen, Steven Sudgen, Christopher Quince, Jeroen H. J. Ensink, Belen Torondel, Alan W. Walker

**Affiliations:** ^1^School of Engineering, University of Glasgow, Glasgow, United Kingdom; ^2^Department of Infection Biology, Faculty of Infectious and Tropical Diseases, London School of Hygiene and Tropical Medicine, London, United Kingdom; ^3^Institute of Biodiversity, Animal Health and Comparative Medicine, University of Glasgow, Glasgow, United Kingdom; ^4^LeAF, Wageningen, Netherlands; ^5^Bear Valley Ventures Ltd., Tarporley, United Kingdom; ^6^Department of Veterinary Medicine, University of Cambridge, Cambridge, United Kingdom; ^7^Ifakara Health Institute, Ifakara, Tanzania; ^8^Hanoi University of Civil Engineering, Hanoi, Vietnam; ^9^Environmental Health Group, Department of Disease Control, Faculty of Infectious and Tropical Diseases, London School of Hygiene and Tropical Medicine, London, United Kingdom; ^10^Organisms and Ecosystems, Earlham Institute, Norwich, United Kingdom; ^11^Gut Microbes and Health, Quadram Institute, Norwich, United Kingdom; ^12^Warwick Medical School, University of Warwick, Coventry, United Kingdom; ^13^Pathogen Genomics, Wellcome Sanger Institute, Hinxton, United Kingdom; ^14^The Rowett Institute, University of Aberdeen, Foresterhill, United Kingdom

**Keywords:** sanitation, microbiota, 16S rRNA gene sequencing, pit latrines, decomposition

## Abstract

Pit latrines are used by billions of people globally, often in developing countries where they provide a low-tech and low-cost sanitation method. However, health and social problems can arise from a lack of emptying or maintenance of these facilities. A better understanding of the biological and environmental parameters within pit latrines could inform attempts to enhance material decomposition rates, and therefore slow fill-up rate. In this study, we have performed a spatial analysis of 35 Tanzanian pit latrines to identify bacteria and environmental factors that are associated with faster or slower pit latrine fill-up rates. Using ordination of microbial community data, we observed a linear gradient in terms of beta diversity with increasing pit latrine sample depth, corresponding to a shift in microbial community structure from gut-associated families in the top layer to environmental- and wastewater-associated taxa at greater depths. We also investigated the bacteria and environmental parameters associated with fill-up rates, and identified pH, volatile solids, and volatile fatty acids as features strongly positively correlated with pit latrine fill-up rates, whereas phosphate was strongly negatively correlated with fill-up rate. A number of pit latrine microbiota taxa were also correlated with fill-up rates. Using a multivariate regression, we identified the *Lactobacillaceae* and *Incertae_Sedis_XIII* taxa as particularly strongly positively and negatively correlated with fill-up rate, respectively. This study therefore increases knowledge of the microbiota within pit latrines, and identifies potentially important bacteria and environmental variables associated with fill-up rates. These new insights may be useful for future studies investigating the decomposition process within pit latrines.

## Introduction

Access to adequate sanitation has a major impact on reducing human illness and death, especially among children (Nakagiri et al., 2016; UNICEF-WHO, 2020). In 2020, ~2 billion people still lacked a basic sanitation service and 494 million of these people practiced open defecation (UNICEF-WHO, 2020). This leaves the communities these people live in vulnerable to water, sanitation, and hygiene (WASH) related diseases.

A pit latrine is an improved sanitation toilet facility that generally consists of a large pit dug into the earth. Some pit latrines can be covered with concrete slabs, and more advanced versions include: pour flush; borehole pit latrines; and odor controlling versions such as the Ventilated Improved (VIP) and Reed Odorless Earth Closet (ROEC; Nakagiri et al., 2016). Pit latrines are used by billions of people, mostly in developing countries, and remain the only viable sanitation option for many communities where they represent a low-cost and low-tech sanitation system (Graham and Polizzotto, 2013; Holm et al., 2016). A key challenge of pit latrine usage is the eventual filling of the pit. Emptying or replacement of pit latrines is costly and the material is hazardous to empty and dispose of Nakagiri et al. (2016) and Farling et al. (2019). How quickly an individual pit latrine will fill is difficult to determine, and is related to latrine size and architecture, the number of users, drainage rate, decomposition rates, and whether other wastes such as household garbage have also been added to the pit.

In our previous work, we characterized the bacterial diversity and composition of 30 pit latrines in Tanzania and Vietnam using high-throughput 16S rRNA gene sequencing (Torondel et al., 2016). We found that the microbial communities within pit latrines were highly dependent on geographical location and user habits, and correlated with environmental factors including pH, temperature, and organic matter content/composition (Torondel et al., 2016). However, in that cross-sectional study we did not address questions related to pit latrine decomposition and fill-up rates. Decomposition rates within pit latrines are highly variable, and dependent on multiple factors such as moisture content, aerobic vs. anaerobic conditions, and the presence of inhibitory compounds (Nwaneri et al., 2008; Van Eekert et al., 2019). The microbial communities within a pit latrine are also of key importance for organic matter degradation. Therefore, optimizing the degrading capacity of the microbiota within these latrines may slow the rate of filling. However, the composition within latrines, or the source of microbial communities, has not been well described in the literature and they are essentially operated as “black box” systems.

The pit latrine environment is not homogenous. Microorganisms will enter the pit from human feces, as well as from the surrounding environment, soil and groundwater. In our previous study (Torondel et al., 2016), we observed 3% operational taxonomic unit (OTU) richness that varied from 173 to 1,903 in individual latrines. These values fell within the range of values typically found in the human gut, which is a relatively low diversity environment, and soil, a comparatively highly diverse microbial community (Turnbaugh et al., 2010; Singh et al., 2014). This supports assumptions that the microbial communities within the pit latrines consist of more microbial taxa than just those originating from human feces. It is generally recognized that the top layer of pit latrines will be more representative of the influent/or fecal content and be subject to oxygen intrusion, while the lower layers will be more anoxic (Van Eekert et al., 2019). Pathogens have been observed to be consistently abundant across pit latrine depth (Capone et al., 2021), but changes in prevailing environmental conditions may impact the overall microbial communities, and decomposition rates, within different regions of the pit. Therefore, the spatial distribution of microorganisms within an individual pit latrine is potentially an important factor to consider. However, the distribution and source of the microbial communities that are most responsible for decomposition or fill-up rates remain largely unknown.

To this end, in this paper we sought to explore the biological and environmental factors affecting the rate of decomposition within pit latrines. Whilst our previous cross-sectional study (Torondel et al., 2016) provided invaluable information about the diversity and composition of bacterial communities found in pit latrines from different geographical locations, in this study our aims were to i) determine if there is spatial variation in bacterial communities within 35 Tanzanian pit latrines at varying sampling depths (sampled over 1 year) and ii) if these profiles could be linked to environmental factors and pit latrine filling rates.

## Materials and methods

### Study area and latrine selection

The study was conducted in Tanzania in the villages of Sululu and Signali. A total of 50 latrines were selected for this study (Supplementary Figure 1a). The details for all the latrines sampled are given in Supplementary Data 1. However, only 35 pit latrines were used in the final microbiota analysis due to challenges with DNA extraction and obtaining sufficient read numbers from the microbial sequencing. Characteristics of latrines used are presented in Supplementary Table 1. Further details regarding the local environment and general pit latrine construction can be found in both Irishet al. (2013) and Torondel et al. (2016).

### Sample collection

Samples were collected from the individual latrines at depth intervals of 20 cm using with a standard soil auger for solid consistency material (Eijkelkamp, Giesbeek, The Netherlands), or with a sterile 150 ml plastic container attached to the soil auger for samples with a liquid consistency. The sampling device is shown in Supplementary Figure 1b. The following environmental parameters: pH, temperature, total and soluble chemical oxygen demand [CODt and CODs], volatile fatty acids [VFAs], total solids [TS], volatile solids [VS], ammonia, total phosphate, carbohydrate and protein were measured as outlined in Torondel et al. (2016).

### Assessment of pit latrine fill-up rates

At the beginning of the study (January 2011), the exact internal dimensions of the pit latrines were determined. Afterwards, changes in the empty pit volume were measured using a digital laser reader every second month over the course of the 1-year sampling period from February 2011 to February 2012 (Supplementary Figure 1c). The fill-up rate (liters/person/day) was then determined using the equations below and the raw data is provided in Supplementary Data 2. Positive fill-up rates imply accumulation or increase of volume and negative fill-up rates vice versa imply decomposition or decrease in volume.


(1) $$\begin{array}{l} \text{ Fillup}\_\text{rate }\left(\frac{\frac{\text{litres}}{\text{person}}}{\text{day}}\right)=\;\\ \frac{\text{Total}\_\text{volume}\_\text{change}\times \text{1000}}{\text{Number}\_\text{days}\_\text{monitored}}\;\times \text{Average}\_\text{users } \end{array}$$

(2) $$\begin{array}{l} \text{ Total}\_\text{volume}\_\text{change }\\ \text{=}\frac{\text{Total}\_\text{depth}\_\text{change}\times \text{Latrine}\_\text{width}\times \text{Latrine}\_\text{length}}{\text{Number}\_\text{days}\_\text{monitored}}\\ \;\times \text{Average}\_\text{users} \end{array}$$

(3) $$\begin{array}{l} \text{ Total}\_\text{depth}\_\text{change }\\ \text{= Depth}\_\text{February}\_\text{2011-Depth}\_\text{February}\_\text{2012} \end{array}$$

### DNA extraction and 454-pyrosequencing

Samples for DNA analysis were kept at −80°C until DNA extraction was performed (in 2012). DNA was extracted from the samples using FastDNA® SPIN Kits for Soil and a FastPrep-24 bead-beading machine (MP Biomedicals, Santa Ana, USA), according to the manufacturer's instructions. Bacterial DNA was amplified using the barcoded primers 338F and 926R according to the protocol described in Torondel et al. (2016). PCR products were cleaned using the Wizard PCR product purification kit (Promega, Fitchburg, Wisconsin, USA) and were then pyrosequenced at the Wellcome Sanger Institute in 2012 using the Lib-L kit on the 454 GS FLX Titanium System (Roche, Branford, Connecticut, USA). Further details on the protocols used to generate 16S rRNA gene sequence data are as described in Torondel et al. (2016).

### Bioinformatics

The sequence data was processed using the AmpliconNoise pipeline for pyrosequencing data (Quince et al., 2009, 2011), during which the samples were demultiplexed, filtered and trimmed (Quince et al., 2009). The filtered flowgrams were clustered to remove errors, and then converted into sequences using the PyroNoise algorithm. They were then further clustered by SeqNoise to remove PCR single base errors. Following these steps, chimeric sequences were then identified and removed using the Perseus algorithm (Quince et al., 2011). The sequences that passed these quality control steps were classified using the RDP classifier (Wang et al., 2007). OTUs were generated using pair-wise Needleman–Wunsch alignment and hierarchical clustering with an average linkage algorithm and a 3% sequence similarity cut-off. We then performed multiple sequence alignment of the OTU consensus sequences using mafft (Katoh and Standley, 2013) and generated a phylogenetic tree using FastTree (Price et al., 2010), which was used in the beta-diversity analysis. Tax4Fun (Aßhauer et al., 2015) was then used to predict the functional capabilities of microbial communities based on 16S rRNA gene datasets after matching sequence taxonomies using the SILVA reference database (Quast et al., 2012). The Kyoto Encyclopedia of Genes and Genomes (KEGG) pathways associated with known prokaryotic organisms are available in Tax4Fun for SILVA SSU Ref NR database release 115 and the KEGG database release 64.0. We used the fctProfiling = FALSE in Tax4Fun() function to recover KEGG pathways (N samples × K KEGG Orthologs) according to the MoP-Pro approach (Aßhaauer and Meinicke, 2013). Although the Tax4Fun-based metabolic predictions are limited by the taxa available in the reference database, it provides a statistic called fraction-of-taxonomic-units-unexplained (FTU), which reflects the number of sequences that are assigned to a taxonomic unit and do not map to organisms or pathways in the KEGG reference database. In our case this value was low, at ~15% and thus, with high matches to the reference database, increases our confidence in our recovered predicted metabolic functions. Despite this, we only used recovered metabolic profiles for diversity analysis and not any downstream differential analysis.

Seqenv (Sinclair et al., 2016) was then used to search OTU sequences against the “nt” nucleotide database by NCBI, textual information on isolation sources of the search results were collated, and a text mining algorithm was used to identify and parse words associated with the Environmental Ontology (EnvO). The normalized frequencies (using DESeq2) of EnvO terms for each OTUs were then multiplied with the OTU table to generate a new table (N samples × E EnvO terms) representing abundances of EnvO terms. The OTU table, EnvO table, phylogenetic tree, and taxonomic information, and meta data were then used in multivariate statistical analysis in the context of environmental parameters.

### Data curation and processing

Out of a total of 50 latrines sampled (at varying depths) a total of 35 individual latrines were used in the microbiome analysis, due to challenges with DNA extractions, PCR and sequencing. The lower layers, in particular, were difficult to extract and sequence. We have only retained samples that had reads >1,000 (*n* = 79). Further to this, in the analysis involving statistics, we have only considered samples with complete environmental data and the top 4 sampling depths, due to the low numbers of samples with sequences from depths beyond 80 cm (*n* = 62). Environmental data consisted of measurements of pH, temperature, total solids (TS), volatile solids (VS), volatile fatty acids (VFA), total chemical oxygen demand (CODt), soluble chemical oxygen demand (CODs), the percentage of soluble chemical oxygen demand to total chemical oxygen demand (%CODs/t), ammonia, phosphate, protein, and carbohydrate. A spreadsheet of the samples and associated metadata from this study are given in Supplementary Data 3.

### Statistical analyses

Statistical analyses were performed in *R* using the tables and data generated as above as well as the meta data associated with the study. For community analysis (including alpha and beta diversity analyses) we used the vegan package (Dixon, 2003). To calculate weighted Unifrac distances (that account for phylogenetic closeness as well as abundance count), we used the phyloseq (Mcmurdie and Holmes, 2013) package. Nonmetric Distance Scaling (NMDS) plot of community data (OTUs at 3% divergence) was performed using the metamds() function from the vegan package. The samples were grouped for different depths as well as the mean ordination value and spread of points [standard errors of the (weighted) averages as ellipses using vegan's ordiellipse() function]. We used adonis() from the vegan package for the analysis of variance i.e., partitioning distance matrices among sources of variation (both qualitative and quantitative information). This function, hitherto referred to as PERMANOVA, fits linear models (e.g., factors, polynomial regression) to distance matrices and uses a permutation test with pseudo-F ratios.

For differential analyses between different pit latrine depths, we used the DESeqDataSetFromMatrix() function from the DESeq2 package (Love et al., 2014) with a significance value cut-off of 0.01. This function allows negative binomial Generalized Linear Model (GLM) fitting (as abundance data from metagenomic sequencing is overdispersed) and Wald statistics for abundance data. After performing multiple testing corrections, it reported families/EnvO terms that varied significantly between depths. Similarly, to find KEGG pathways that were significantly different between pit latrine sampling depths, we used the Kruskal–Wallis test with *p*-values adjusted for multiple comparisons using the Benjamini–Hochberg procedure. We also calculated Local Contributions to Beta Diversity (LCBD) (Legendre and Cáceres, 2013) where the total beta diversity is calculated as the variance of the community data and is then broken up into sample-wise contributions. We followed the procedure where a Hellinger transform is first applied to the abundance data (Samples × Families) to calculate a *squared differences from column mean* table from where sum of columns gave the LCBD values of the samples.

For regression analysis of fill-up rate against families and environmental data (Table 1 and Supplementary Data 4), we used the car package in R (Fox and Weisberg, 2010) for regression diagnostics; the gvlma package (Peña and Slate, 2015) for checking linear model assumptions; a customized script (Ijaz, 2018) to use variance inflation factor (VIF) to remove collinearities; the step() function for formula-based model filtering using Akaike Information Criteria (AIC); the leap package (Lumley and Miller, 2009) to perform subset regression; and the DAAG package (Maindonald and Braun, 2015) to perform cross-validation for linear regression.

**Table 1:** Fit of environmental parameters to pit latrine fill-up rate using linear regression models.

**Methods**	**Adjusted R^2^**	**Mean Squared Error (MSE) - cross-validation**	β**-coefficients**										
			**Intercept**	**pH**	**Temperature**	**VS**	**VFA**	**CODs**	**CODt**	**%CODs/ CODt**	**Ammonia**	**Phosphate**	**Carbohydrate**
Model 1:	0.141	0.142	1.088741 (*p* = 0.352)	0.198289 (*p* = 0.023*)	−0.003043 (*p* = 0.932)	0.004039 (*p* = 0.174)	0.002237 (*p* = 0.242)	0.000422 (*p* = 0.652)		0.001529 (*p* = 0.889)	−0.007380 (*p* = 0.264)	−0.03180 (*p* = 0.037 *)	−0.000257 (*p* = 0.379)
Model 2:	0.599	0.086	1.489984 (*p* = 0.15174)	0.324684 (*p* = 0.00030***)	−0.020371 (*p* = 0.51663)	0.006578 (*p* = 0.00867**)	0.004985 (*p* = 0.06218)	0.000873 (*p* = 0.22698)		0.000617 (*p* = 0.94248)	−0.007640 (*p* = 0.29085)	−0.062989 (*p* = 0.00064***)	−0.000237 (*p* = 0.32700)
Model 3:	0.638	0.074	−2.088131 (*p* = 0.0004***)	0.327766 (*p* = 0.0001***)		0.007068 (*p* = 0.0023**)	0.005878 (*p* = 0.0070**)	0.000759 (*p* = 0.2231)			−0.009810 (*p* = 0.1176)	−0.066625 (*p* = 7.4e−05***)	−0.000243 (*p* = 0.2537)
Model 4:	0.627	0.071	−2.142545 (*p* = 0.00048***)	0.336903 (*p* = 0.00014***)		0.008364 (*p* = 0.00614**)	0.006498 (*p* = 0.00745**)	0.001305 (*p* = 0.18395)	−0.000173 (*p* = 0.45394)		−0.011337 (*p* = 0.09465)	−0.068688 (*p* = 0.00010 ***)	−0.000316 (*p* = 0.18860)

Predictors that may contribute to the fill-up rate are shaded as red (e.g., high pH), and those that may cause the latrines to fill-up more slowly are shaded as blue (e.g., addition of phosphate). If the explanatory variable was not selected in the method, the cell is empty.

In Table 1, we show the results after fitting the linear equation, *Y*_*i*_ = β_0_ + β_1_ *X*_1i_ + β_2_ *X*_2i_ + β_3_ *X*_3i_ + ε_*i*_, on environmental covariates from pit latrines. Here Y_i_ is the fill-up rate for top layer (depth 1) to give *n* = 25 samples. The explanatory data X_i_ are highlighted as bold in the table. Before applying the linear regression, we tested for homoscedasticity (constant variance) to make sure the linear assumptions held. For this purpose, we used the Breusch–Pagan (BP) test, or the Lagrange multiplier test for heteroscedasticity, with the null hypothesis being the residuals were homoscedastic. The BP test (χ^2^ = 0.034, *p* = 0.8532) suggested to use the linear regression model. We gained additional confirmation by employing gvlma() from R's gvlma package, which also suggested that heteroscedasticity was not satisfied. We then chose several methods: Method 1, in this method we used the variance inflation factor (VIF) to remove collinear terms from the environmental parameter by using a threshold of 10. TS, CODt, and Protein were removed in this case. We then performed the regression of fill-up rate against the environmental data; Method 2, after removing colinear terms and performing regression, we then used regression diagnostics (R's car package) to identify extreme observations using the Bonferonni *p*-value test to remove T28 (*p* = 0.94714) as well as T27 and T33, which were removed based on Cook's distance; Method 3, we used step-wise regression (without employing VIF to remove any colinear terms) using AIC on all the samples minus T28, T27, and T33; and Method 4, we used the subset regression after removing T28, T27, and T33. For each model, we provided adjusted *R*^2^ as quality of fit. Furthermore, we performed cross-validation for linear regression using 10 folds in the CVlm() function from R's DAAG package. We used 10 folds cross-validation where we randomly dropped 10 samples and trained the regression model on the remaining data to get an additional Mean Squared Error (MSE), which accounts for any outliers in the dataset.

For each statistical method, appropriate normalization was used. For example, where we used alpha-diversity estimates and regression analysis, we rarefied the abundance table to the lowest library size. DESeq2 for differential analysis uses its own median of ratios normalization where abundance count is divided by sample-specific size factors determined by the median ratio of amplicon counts relative to geometric mean per amplicon. To visualize the results from DESeq2, the expression levels were drawn after using log-relative normalization on the abundance tables (Ijaz et al., 2017).

The statistical scripts and workflows for all of the methods above can be found at http://userweb.eng.gla.ac.uk/umer.ijaz#bioinformatics.

## Results

**Figure 1:**
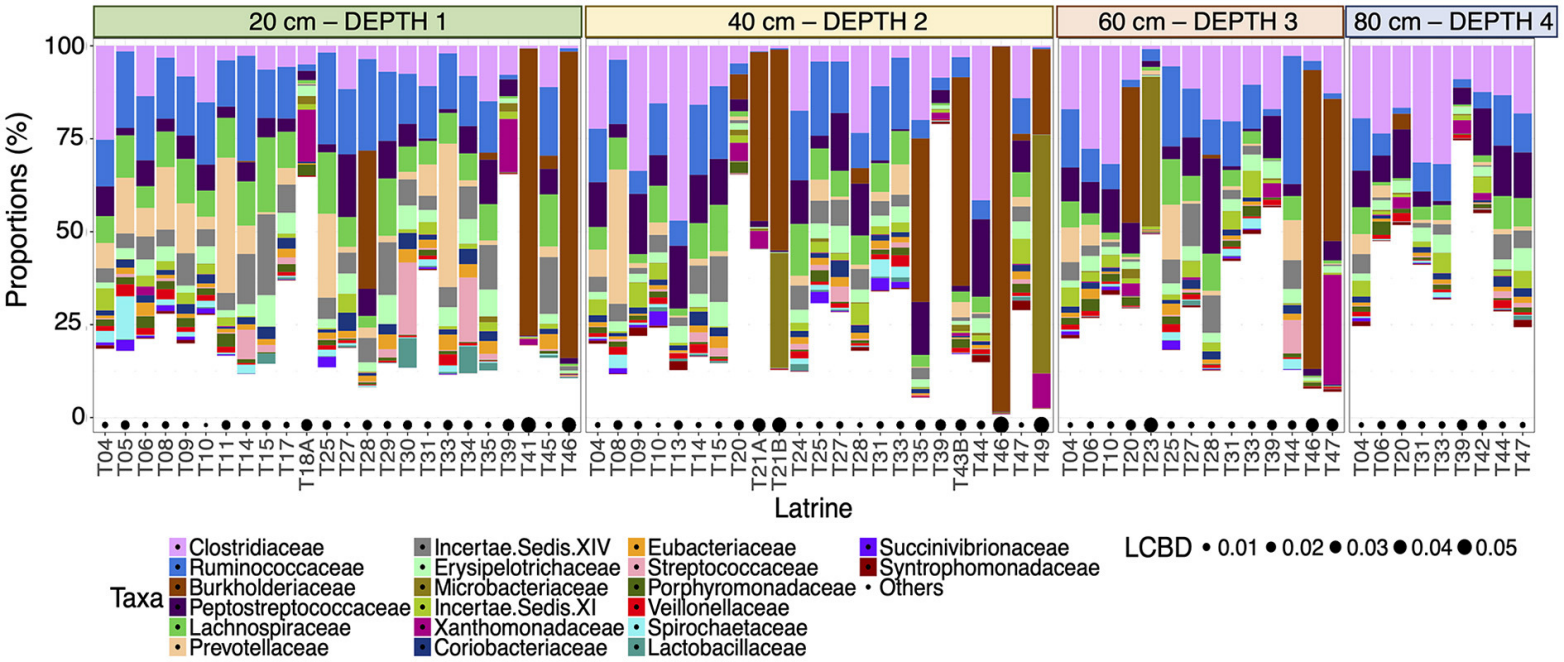
Stacked bar plot of the 20 most proportionally abundant family-level taxa (on the y-axis as percentage relative proportion) for all the samples from individual pit latrines (labeled T##) ordered by depth from 20 (Depth 1), 40 (Depth 2), 60 (Depth 3), and 80 cm (Depth 4) [left to right on the x-axis]. Local Contributions to Beta Diversity (LCBD) values are drawn as bubbles below. A higher LCBD value suggests the community is different from the average community profile (indicating outliers).

We characterized the microbial communities within the pit latrines and found that the most abundant taxa were relatively similar for individual pits at the same sampling depth (Figure 1). *Clostridiaceae* and *Ruminococcaceae* were observed to be the two most proportionally abundant families out of the top 20 observed families (Figure 1). Local contribution to beta diversity (LCBD) values varied between latrines, but not across different depths within the same latrine (Figure 1), indicating that individual latrines are a greater source of microbiota variation than depth.

**Figure 2:**
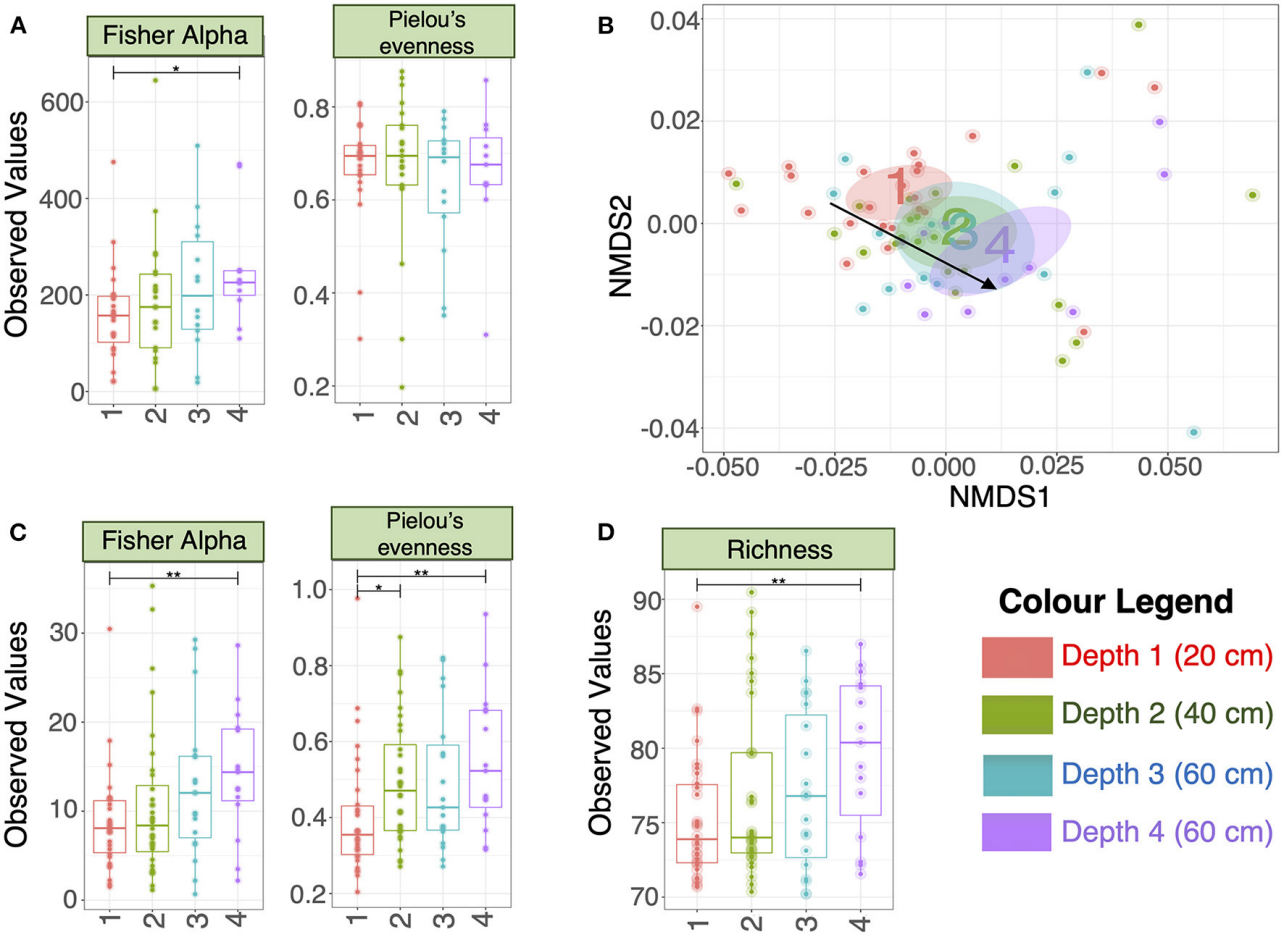
**(A)** Alpha diversity metrics calculated on the rarefied microbiota data (OTUs at 3% divergence) from the four different pit latrine sample depths (Depth 1 = 20 cm, Depth 2 = 40 cm, Depth 3 = 60 cm, and Depth 4 = 80 cm); **(B)** Nonmetric multidimensional distance scaling (NMDS) plot (beta diversity) using weighted Unifrac distance. The ellipses represent the 95% confidence interval of the standard error of the ordination points of a given category (Depth 1 = 20 cm, Depth 2 = 40 cm, Depth 3 = 60 cm, and Depth 4 = 80 cm) and the labels are drawn at the mean value of the ordination points; **(C)** Alpha diversity metrics calculated on the rarefied EnvO table returned from seqenv pipeline, indicating that the diversity of defined “descriptors” increased with increasing pit sample depth (Depth 1 = 20 cm, Depth 2 = 40 cm, Depth 3 = 60 cm, and Depth 4 = 80 cm); **(D)** Richness was calculated as the exponential of Shannon entropy on the proportional representation of 284 KEGG pathways returned from Tax4Fun software. Depth 1 = 20 cm, Depth 2 = 40 cm, Depth 3 = 60 cm, and Depth 4 = 80 cm. For **(A,C,D)**, we performed pair-wise ANOVA, taking two pit latrine sampling depths at a time. Significance values were indicated as follows (*0.01 ≤ *p* < 0.05; **0.05 ≤ *p* < 0.001. Other alpha-diversity measures (Richness, Shannon and Simpson) are shown in Supplementary Figure 2.

**Figure 3:**
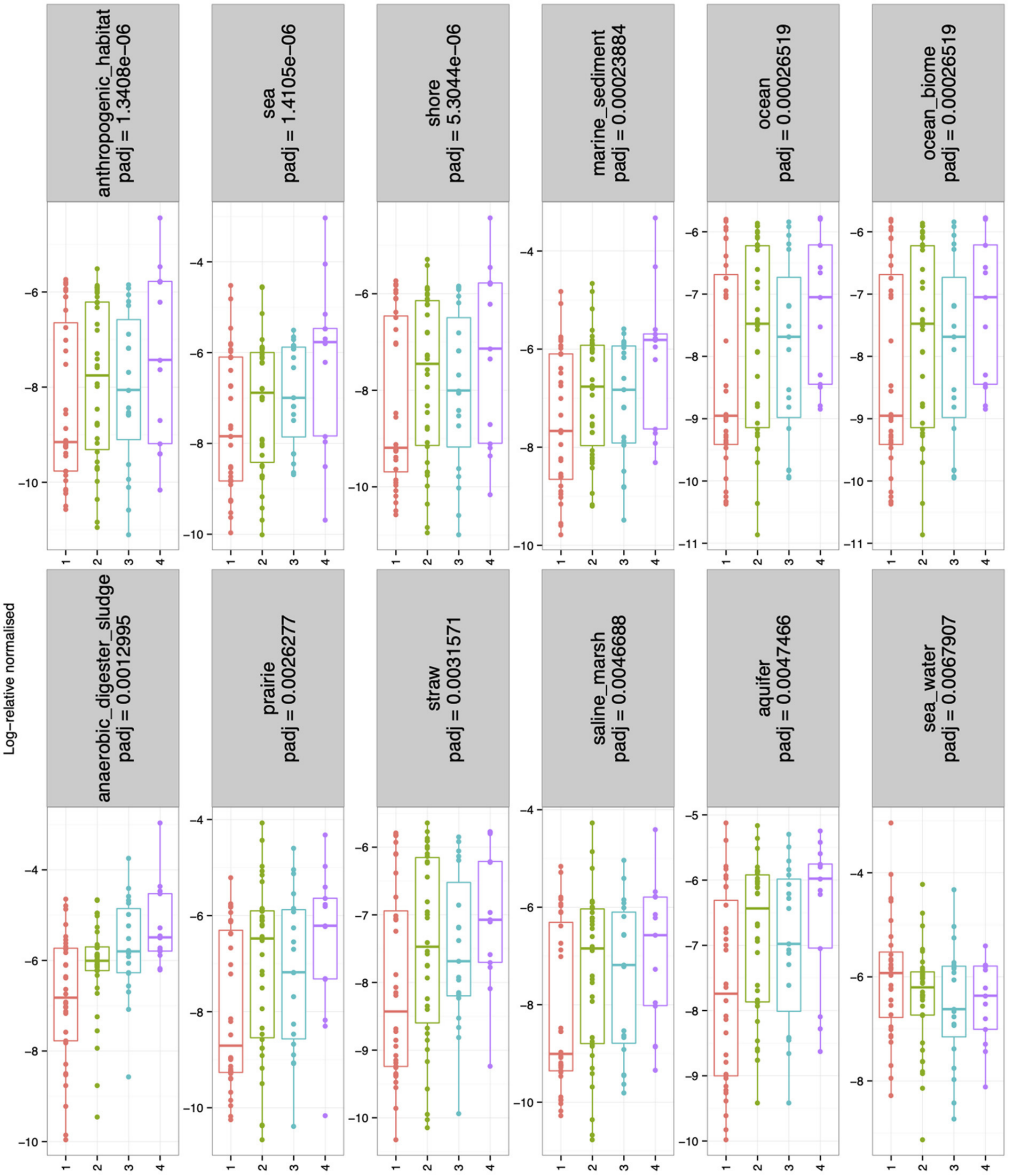
Boxplots showing the environmental ontology (EnvO) terms returned from the seqenv pipeline that were log-2 fold differentially abundant for the combined pit latrine samples at sampling depths 1, 2, 3, and 4. Depth 1 = 20 cm, Depth 2 = 40 cm, Depth 3 = 60 cm, and Depth 4 = 80 cm.

There were no significant differences in alpha-diversity (Evenness, Richness, Shannon and Simpson diversity indices) between the four sampled pit depth layers (Figure 2A). However, when we analyzed beta diversity estimates we observed that with increased pit sample depth there was less dissimilarity, or more overlap in the microbial community composition (Figure 2B). Next, we considered microbiota diversity with respect to Environmental Ontology (EnvO; https://sites.google.com/site/environmentontology/) terms returned from the seqenv pipeline. EnvO provides a unified metadata annotation from deposited sequences from The National Center for Biotechnology Information (NCBI). It collates descriptions of environmental information and where sequences are isolated from. This pipeline may therefore allow us to elucidate the origin of microbial community members in the pit latrines, and how these might change with pit sample depth. We observed differences in all alpha-diversity measures with respect to depth and the terms recovered from “isolation source” (287 unique terms), with the number of unique terms increasing with increased pit sample depth (Figure 2C). When looking at the richness of the predicted pathways (Figure 2D) we observed an increase in the number of KEGG pathways identified as pit sample depth increased. There were a total of 12 EnvO terms that were differentially abundant at different depths. We observed an increase in “anaerobic digester sludge” and “anthropogenic habitat” terms with increasing pit sample depth (Figure 3). Other identified terms include various environmental habitats such as “sea_water”, “ocean”, “prairie”, “aquifer”, and “saline_marsh”, which in general also increased with increased pit sample depth. This indicates that the microbial populations were indeed changing with respect to pit latrine sampling depth.

**Figure 4:**
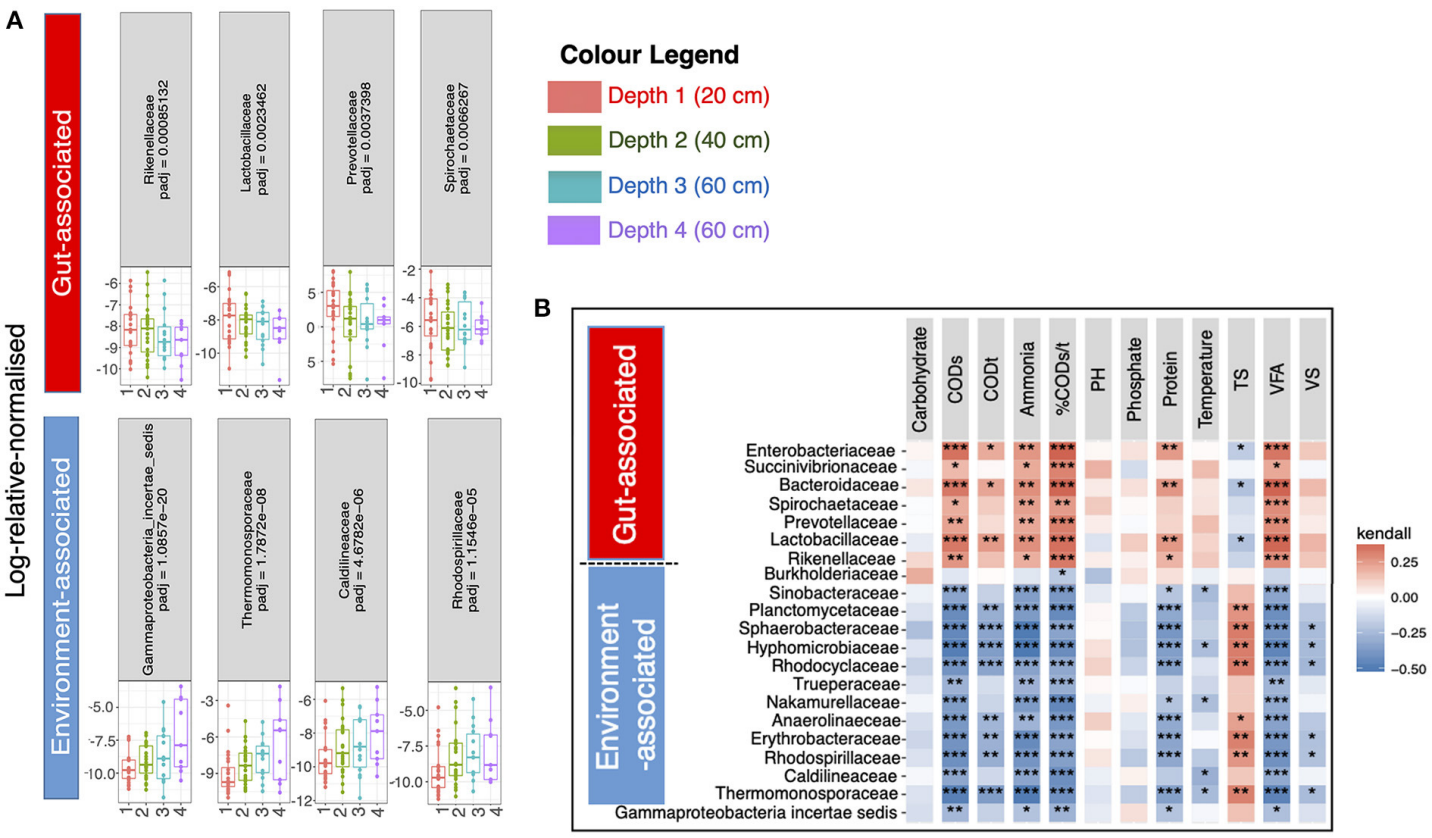
21 families were significantly different (Adjusted *p*-values ≤ 0.05) between the four pit latrine sample depth layers (Depth 1 = 20 cm, Depth 2 = 40 cm, Depth 3 = 60 cm, and Depth 4 = 80 cm). In **(A)** four families that were predominantly gut-associated species (*n* = 7) are drawn in the top row whilst the families that increased in proportional abundance with greater depth, and four families that were predominantly environment-associated, (*n* = 14) are drawn in the bottom row. The remaining familes are shown in Supplementary Figure 3. Correlation plots **(B)** of the full 21 families for samples at Depth 1 (20 cm), Depth 2 (20 cm), Depth 3 (60 cm), and Depth 4 (80 cm) with the recorded environmental factors are drawn top right. For this calculation we took the relative proportions of the abundance data before calculating the Kendall correlation. The *p*-values for a single environmental factor (column) were adjusted for multiple tests using the Benjamini and Hochberg (1995) correction method. The adjusted significant *p*-values are represented as *0.01 ≤ *p* < 0.05; **0.05 ≤ *p* < 0.001; and ***p ≤ 0.001 in top right panel. Depth 1 = 20 cm, Depth 2 = 40 cm, Depth 3 = 60 cm, and Depth 4 = 80 cm.

Next, we wanted to determine which specific microbiota taxa were changing with respect to pit latrine sample depth. To do this we performed regression analysis at the family level (208 families in total). The primary reason for collating data at the family level, and then employing it in the regression modeling, was to avoid the linear dependencies between taxa, which are higher at lower levels (genera and species) and can thereby adversely affect the regression performance (and hence the reason why variance inflation factor is suggested in our analyses). A total of 21 families were differentially abundant across pit latrine sample depth (Figure 4). Families that were more proportionally abundant in the top pit sample layers were *Rikenellaceae, Lactobacillaceae, Burkholderiaceae, Prevotellaceae, Spirochaetaceae, Bacteroidaceae, Succinivibrionaceae*, and *Enterobacteriaceae*. With the exception of *Burkholderiaceae*, which would be considered as environmental-associated, these families are all associated with the human gut. In contrast, families with increased proportional abundance with increasing pit sample depth were *Gammaproteobacteria_incertae_sedis, Thermomonosporaceae, Caldilineaceae, Rhodospirillaceae, Erythrobacteraceae, Anaerolineaceae, Nakamurellaceae, Trueperaceae, Rhodocyclaceae, Hyphomicrobiaceae, Sphaerobacteraceae, Plactomycetaceae*, and *Sinobacteraceae*. These are typically environment-associated taxa and wastewater associated. Thus, as expected, our data indicate that the top layers of pit latrines are dominated by human fecal-associated bacteria, while environmental taxa become more proportionally dominant at lower depths.

**Figure 5:**
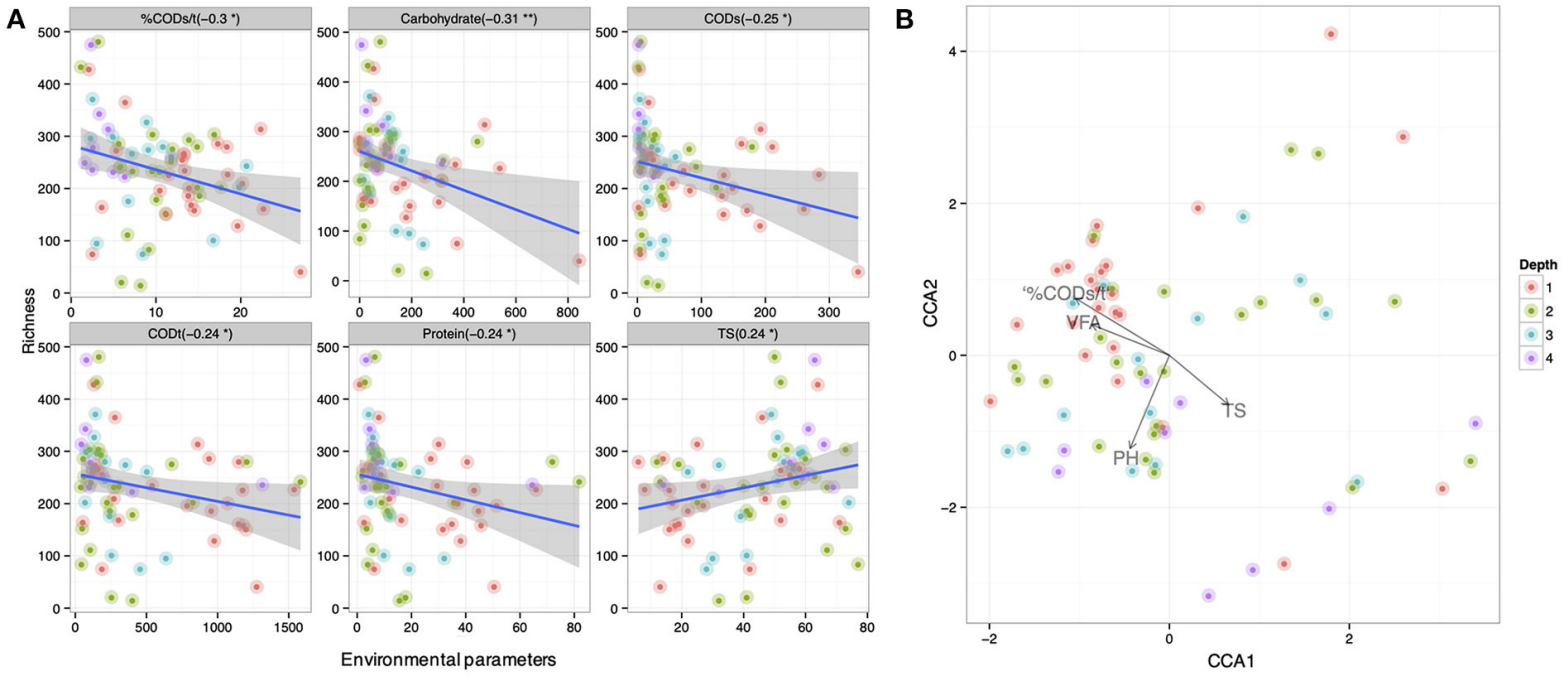
**(A)** Correlation between richness of samples at depth 1, 2, 3, and 4 (70 samples with 14,803 OTUs; and colored by depth) and the environmental parameters. Here only the significant parameters are shown. **(B)** Canonical correspondence analysis of community data against significant environmental parameters (based on PERMANOVA) for samples at depths 1, 2, 3, and 4. Depth 1 = 20 cm, Depth 2 = 40 cm, Depth 3 = 60 cm, and Depth 4 = 80 cm.

Variation in pit latrine layers may be explained by the variation of environmental conditions. Therefore, we next wanted to correlate microbial community structure from the varying sampling depths with the environmental parameters. We observed that microbial community richness was correlated with numerous environmental parameters across pit latrine sample depths (Figure 5A). Within this, we found that the CODs, CODt, carbohydrate, and protein concentrations and the %CODs/t ratio were all negatively correlated with the richness of the bacterial community (i.e., increased concentrations led to reduced diversity). In contrast, total solids (TS) concentrations were positively correlated with increased richness. In these plots, typically samples from depth 1 clustered together and were distinct from the samples at other depths. For example, in the environmental plots for CODt and protein parameters, samples from depth 1 clustered toward increased concentrations and richness values ~300 (Figure 5A). When looking at individual environmental factors, we identified Latrines (accounting for 3.37% variability; *p*-value 0.003), Depth (accounting for 2.87% variability; *p*-value 0.005), pH (accounting for 3.03% variability; *p*-value 0.004), TS (accounting for 3.91% variability; *p*-value 0.001), VFA (accounting for 2.17% variability; *p*-value 0.038), CODt (accounting for 2.44% variability; *p*-value 0.015), %CODs/t (accounting for 2.88% variability; *p*-value 0.004), and Phosphate (accounting for 2.27% variability; *p*-value 0.029) as the significant factors correlated with the microbial community composition (by performing PERMANOVA on the community data). We then performed regression of richness as well as constrained ordination (CCA) of the microbial community data against the environmental variables (Figure 5B). From this, we observed that a positive environmental gradient existed with VFA and %CODs/t (clustered with upper sampling depths) and a negative gradient was associated with TS. These results highlight the impact of environmental parameters on pit latrine microbial richness, with COD, proteins, VFAs, and solids potentially important parameters to consider.

Finally, in order to investigate why different pit latrines fill at varying rates, we correlated microbiota structure with latrine environmental parameters and fill-up rates. Fill-up rates are ultimately the most important aspect in pit latrine functioning, with a completely filled pit requiring emptying or replacement. We focused on the samples from the top layer (Depth 1), for which OTUs assigned at family level with environmental and fill-up data were available (25 samples). This stringent sampling strategy was necessary to enable us to correlate both the environmental variables and the community structure with the fill-up rate. The environment strongly correlated with the microbiota profiles, explaining just over 30% of the variability in community structure at the family level. The previous results (Figure 5A) and PERMANOVA values highlighted the strong influence of environmental parameters (e.g., pH, COD, VFA, phosphate, and carbohydrates). For this reason, we correlated the environmental variables with fill-up rate independent of community structure. Once we removed three outliers, a significant multivariate fit of fill-rate as a function of the environmental variables was obtained (adj. *R*^2^ = 0.638). From this, VFAs, VS, and pH were all strongly positively associated with fill-up rates (Table 1). Of these, the most significant variable was pH (i.e., higher pH correlated with faster fill-up rates). On the other hand, phosphate was strongly negatively correlated with fill-up rate (i.e., pits filled up slower with increasing phosphate).

Next, we investigated whether the microbial community structure could have an influence on fill-up rate beyond that variation that was accounted for by the environmental variables described above. We did this by adding the 20 most abundant bacterial family-level taxa to our multivariate regression (Supplementary Data 4) and detected the subset of taxa and environmental variables that best correlated with fill-up rate. The overall fit increased from adj. *R*^2^ = 0.638 to adj. *R*^2^ = 0.79, suggesting that the microbiota does indeed contribute to the fill up rate. Taxa from the following families: *Porphyromonadaceae, Lactobacillaceae, Clostridiaceae, Prevotellaceae*, and *Enterobacteriaceae* were positively associated with fill-up rate. In contrast, the *Veillonellaceae, Spirochaetaceae, Incertae_Sedis_XIV, Erysipelotrichaceae, Incertae_Sedis_XI*, and *Incertae_Sedis_XIII* taxa were negatively associated with fill-up rate. The most significant overall fit included two families in addition to the environmental variables, *Lactobacillaceae*, and *Incertae_Sedis_XIII*, which were strongly positively and negatively correlated with fill-up rate respectively.

## Discussion

Pit latrines are widely adopted as improved sanitation systems. One of the main challenges of their use is the rate at which a pit will completely fill, which is both costly and hazardous to remediate. As microbial communities within the pit carry out the decomposition of organic material, it is possible that the longevity of a latrine can be extended if the microbial degradative activity is optimized. However, there is widespread variability in pit latrine filling and decomposition rates, which are intrinsically linked to a variety of external factors such as the number and toilet habits of users, presence of household waste, anaerobic conditions, and the surrounding soil and groundwater. This is important from a public health perspective given that the pits either need to be emptied after filling, or become unusable, potentially leading to increased defaecation in the open. This risks potential increased exposure to diarrhoeal and other pathogens (Farling et al., 2019). In this study, we therefore aimed to better understand how pit latrine microbiota profiles might be linked to pit fill-up rates by determining how the bacterial profiles changed with pit sample depth and if these profiles could be correlated with filling rates and environmental conditions in the pit. We observed clear differences in bacterial communities across pit latrine sample depth and were able to correlate specific microbial taxa with environmental parameters and filling rates.

Microbial pit latrine research has primarily focused on assessing water contamination downstream (Ndoziya et al., 2019; Usman and Aliyu, 2020), and few studies have focused on the microbial communities within the pit latrines. Thus, the microbial communities within pit latrines are hugely understudied and knowledge gaps remain with respect to spatial, temporal, and environmental drivers of microbiota composition and activity. The dominant microbial taxa from the pit latrines in this study were the *Clostridiaceae* and *Ruminococcaceae* families, which we previously also showed were more abundant in Tanzanian pit latrines than in Vietnamese comparator pits (Torondel et al., 2016). These general findings differ from those found in the limited number of other pit latrine microbiome studies. For example, Byrne et al. (2017) observed *Bacteroidales* and *Porphyromonadaceae* as the dominant taxa in pour-flush pit latrines in South Africa, while Beukes (2019) found *Pseudomonas* and *Bacillus* species as the dominant taxa in ventilated improved pit latrines also from South Africa. While geographic and user population differences may explain some of these observed discrepancies, both South African latrine types will also have significant oxygen intrusion, which would impact the latrine microbiome, particularly if anaerobic digestion is typically the primary degradation process (Van Eekert et al., 2019). Nonetheless, it is clear that there will be significant variations in microbiota composition in pit latrines around the world, and that local environmental conditions will be major drivers of microbial community assembly. The fact that our work was focused solely on one region of Tanzania is therefore a limitation of the study, as conclusions from these pit latrines may not be generalized to other locations around the world where pit latrines are commonly used. Moreover, we did not classify the water content of the latrines which may have influenced community composition, or the addition of solid/household waste to the latrines, and whether this might also impact fill rate. Users reportedly did not dispose of solid waste in the latrines, as they are aware of the contribution of solid material to the rapid filling of the pits. However, we did notice menstrual cloths, other cloth-like materials and stones in a small number of latrines, which may be important features to investigate in future work. Another limitation of the study is that we used 454-pyrosequencing, which has reduced throughput compared to the more widely used Illumina-based technology, and suffers from systematic biases including homopolymer errors. Nonetheless, we have attempted to optimize the quality of our data by setting a minimum read depth threshold, which resulted in the drop out of some samples after denoising steps. As a result, the remaining samples that passed our stringent quality control criteria show consistent patterns, and can therefore be deemed to be robust.

In the context of Tanzania, we showed that pit latrine sample depth was an important determinant of microbial community structure, with variation observed in beta diversity dissimilarity, and abundant microbial families, at different depths within latrines. Based on differential expression analysis, we identified families whose dominance was altered significantly while moving down through the pit latrine layers. Many of these families were gut-associated (e.g., *Prevotellaceae*) and decreased in dominance with greater sample depth. In contrast, several environmental-associated families showed the opposite pattern, whereby they increased in predominance with increased sampling depth. This supports the assumption that the top layer of the pit latrine is likely to be much higher in fecal matter (gut-associated), while the lower layers are progressively less favorable for gut-derived microbes and are instead associated with organisms that are ubiquitous to the surrounding environment (Nakagiri et al., 2016; Van Eekert et al., 2019). This was also evidenced by the linear trend in the NMDS plot and the increasing trend of EnvO terms related to “*anaerobic_sludge_digester*” with increasing pit latrine depth. This contrasts with work by Capone et al. (2021) who noted enteric pathogen detection was largely consistent across pit latrine sampling depth. Of note, targeted qPCR was used for enteric pathogen detection in that work, so the wider microbiota was not studied. It is therefore not possible to determine whether or not the observed consistent pathogen detection also occurred in parallel with consistent overall microbiota composition in that study. Regardless, pit latrine configuration may have played a key role as Capone et al. (2021) studied lined pit latrines (just 26% of the latrines in our study were lined—Supplementary Table 1), which may have minimized interactions with the surrounding environmental microbial communities. Future work to link pit latrine configuration with microbial community structure would also help to advance our understanding of these systems.

A further key aim of our study was to correlate microbiota composition and measured environmental variables with pit fill-up rates. Volatile solids (VS) were positively correlated with fill-up rate. This seems intuitive, as the decomposition of fecal matter through anaerobic digestion or decomposition should reduce VS, therefore, if VS is increasing, then fill-up rates may also increase. This would likely be the case if decomposition was lower than the rate at which new organic material was added to the pit. We also observed that pH was strongly positively correlated with fill-up rate (i.e., as pH increased fill-up rate also increased). The correlation with pH is to some extent expected, as a lower pH is indicative of increased microbial degradative activity due to the release of volatile fatty acids (VFA) during anaerobic fermentation (Van Eekert et al., 2019). Increased fermentative activity might therefore result in a lowering of the fill-up rate. However, it is important to note that VFA levels were also positively correlated in this study with fill-up rate. This apparent contradiction could in part be explained by the fact that the accumulation of VFAs (for example acetic and lactic acid) can inhibit various microbial groups, and might therefore subsequently negatively impact decomposition (Wang et al., 1999). Phosphate was negatively correlated with fill-up rate. The correlation with phosphate concentrations is intriguing as it suggests that phosphate may be the limiting nutrient for microbial degradation and, hence, addition of phosphate may be a possible strategy to decrease latrine fill-up rate. Kaspari et al. (2008) analyzed the relative effects of eight nutrient elements on litter decomposition and found that phosphate availability is one of crucial factors to enhance litter decomposition in tropical forests (Kaspari et al., 2008). Different research groups have previously attempted to alter decomposition rates by either addition of a mixture of microorganisms and/or enzymes (Taljaard et al., 2005; Nakagiri et al., 2016). However, Buckley et al. determined no correlation between the use of additives and the rate of change in pit matter content (Buckley et al., 2008). Further investigations are clearly required to elucidate the potential impact of these variables.

We also addressed the question of whether the microbial community structure could influence fill-up rate beyond that variation that is accounted for by the environmental variables. Our results indicate that microbial composition is indeed correlated. *Porphyromonadaceae, Lactobacillaceae, Clostridiaceae, Prevotellaceae*, and *Enterobacteriaceae* were all strongly positively associated with fill-up rate. This finding may be partly explained by the top layers of the pit latrine being continually enriched with these gut-associated microorganisms, hence heavily-used latrines that fill up more quickly could have a higher proportional abundance of fecal-derived microbes. Of note, *Lactobacillaceae* were particularly strongly correlated with pit latrine fill-up rates. The constituent species of this family (e.g., *Lactobacillus, Pediococcus*, and *Paralactobacillus*) typically generate lactic acid as a main fermentation product, which could play an important role in pit latrine microbiota dynamics given that lactic acid has been shown to be a key disruptor of microbial community structure and activity in other related anaerobic environments such as the mammalian gut (Louis et al., 2022). Intriguingly, *Veillonellaceae*, a family that includes many lactic acid-utilizing species (Louis et al., 2022), was negatively associated with pit fill-up rates, adding further circumstantial evidence that lactic acid production/consumption might be important for pit latrine microbiota functionality. The *Incertae_Sedis_XIII* taxon was also strongly negatively associated with fill-up rate. These bacteria are from the class *Clostridia* and are commonly found in the gut, but are poorly characterized and not well understood. Thus, further analysis into the profiles of these organisms, and their interactions within pit latrines may help to unravel these factors.

To conclude, we have shown a clear sample depth gradient within pit latrines, with gut-derived microbes associated with the upper layers and environmental-associated microbes with the lower layers, thus demonstrating that spatial sampling is key for understanding the processes and microbial activity within pit latrines. Additionally, we have identified that pH, VS, VFA, and phosphate were key parameters correlated with pit latrine fill-up rate. We also observed potentially important microbes that were correlated with fill-up rates, however, further controlled experimentation would be required to unravel these interactions. Overall, our work provides valuable novel insight into the microbial communities in these improved sanitation systems, which may be informative for future decomposition-based trials to improve pit latrine performance.

## Data availability statement

The datasets presented in this study can be found in online repositories. The names of the repository/repositories and accession number(s) can be found below: https://www.ebi.ac.uk/ena/browser/view/PRJEB27388. This data is also available at https://github.com/umerijaz/pitlatrines.

## Author contributions

UI, ME, WG, CQ, JE, BT, and AW contributed to the study design. BT managed the study. BT, JE, SS, FA, BL, and V-AN performed the sample collection and analysis test in the field laboratories. BT and OG performed the DNA extraction and PCR. AW and JP performed the 454-sequencing at the Wellcome Sanger Institute. UI performed the bioinformatics and statistical analysis. UI and CK prepared figures. UI, OG, CK, BT, and AW contributed to data interpretation and drafted the paper. All authors contributed to redrafting. All authors (except JE) approved the final manuscript.

## Funding

This research received financial support from the Bill and Melinda Gates Foundation (grant number OPP52641 to the London School of Hygiene and Tropical Medicine). AW and JP were supported by the Wellcome Trust (grant number 098051). AW and the Rowett Institute, University of Aberdeen, receive core funding support from the Scottish Government Rural and Environmental Science and Analysis Service (RESAS). UI is funded by NERC Independent Research Fellowship (NE/L011956/1) and further supported by EPSRC (EP/P029329/1 and EP/V030515/1). CQ is funded through an MRC fellowship (MR/M50161X/1) as part of the MRC Cloud Infrastructure for Microbial Bioinformatics consortium (MR/L015080/1).

## Dedication

This paper is dedicated to our dear friend and colleague Jeroen Ensink (1974-2015), in recognition of his contribution to the sanitation field, and the much valued time he spent with the “pit latrine team” members.

## Conflict of interest

Author WG was employed by Bear Valley Ventures Ltd.

The remaining authors declare that the research was conducted in the absence of any commercial or financial relationships that could be construed as a potential conflict of interest.

## Publisher's note

All claims expressed in this article are solely those of the authors and do not necessarily represent those of their affiliated organizations, or those of the publisher, the editors and the reviewers. Any product that may be evaluated in this article, or claim that may be made by its manufacturer, is not guaranteed or endorsed by the publisher.

## Supplementary material

SUPPLEMENTARY FIGURE S1 **(a)** Example of pit latrine from Tanzania, **(b)** Sampling device to acquire liquid material from pit latrines, and **(c)** method for measuring fill-up rate.

SUPPLEMENTARY FIGURE S2 **(A)** Alpha diversity metrics calculated on the rarefied microbiota data (OTUs at 3% divergence) from the four different pit latrine sample depths (Depth 1 = 20 cm, Depth 2 = 40 cm, Depth 3 = 60 cm, and Depth 4 = 80 cm); **(B)** Alpha diversity metrics calculated on the rarefied EnvO table returned from seqenv pipeline, indicating that the diversity of defined “descriptors” increased with increasing pit sample depth (Depth 1 = 20 cm, Depth 2 = 40 cm, Depth 3 = 60 cm, and Depth 4 = 80 cm).

SUPPLEMENTARY FIGURE S3 Differentially abundant families not shown in Figure 4.

SUPPLEMENTARY DATA S1 Spreadsheet of the characteristics of all the pit latrines sampled in Tanzania.

SUPPLEMENTARY DATA S2 Spreadsheet of the calculation of fill-up rate.

SUPPLEMENTARY DATA S3 Spreadsheet of the samples and associated metadata from this study.

SUPPLEMENTARY DATA S4 Subset regression to address whether the microbial community structure could have an influence on fill-up rate, beyond variation which is accounted for by the environmental variables. In addition to the predictors considered in Table 1, we added the 20 most abundant families to our multivariate regression (after using log-relative transformation of the abundance data in the regression model) using subset regression (Model 4 in Table 1). The top 10 subsets ranked by adjusted R^2^ are given in the table including MSE between cross-validated prediction and the predicted values using all the observations. Similar to Table 1, predictors that may contribute to the fill-up rate are shaded as orange, and those that may cause the latrines to fill-up more slowly are shaded as blue. For each family we also display the genera in parenthesis that were detected for these families in our samples. If the explanatory variable was not selected in the subset, the cell is empty.

SUPPLEMENTARY TABLE S1 Characteristics of the 35 latrines from Tanzania used in the microbial analysis in this study.
