# Supplementary figures and images for: Analysis of pit latrine microbiota reveals depth-related variation in composition, and key parameters and taxa associated with latrine fill-up rate
Source: Front Microbiol. 2022 Sep 23;13:960747. doi: 10.3389/fmicb.2022.960747 (PMC9539666; doi:10.3389/fmicb.2022.960747)

a)

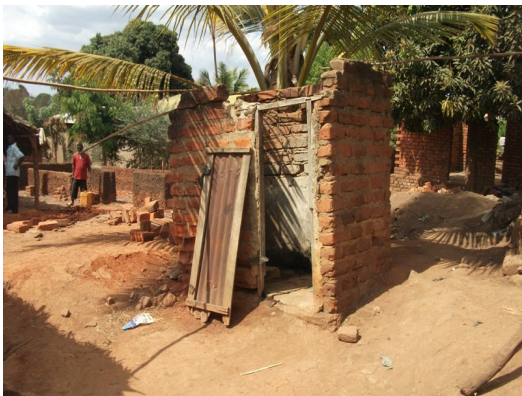

b)

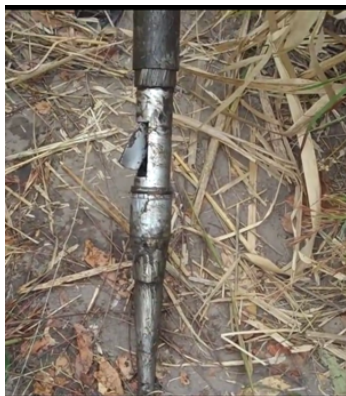

c)

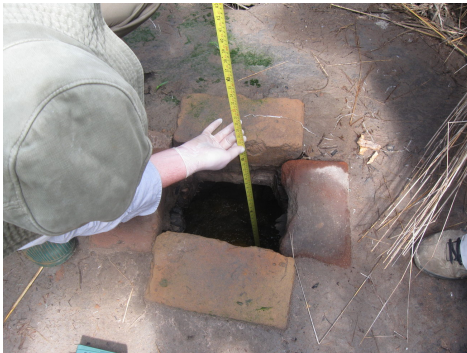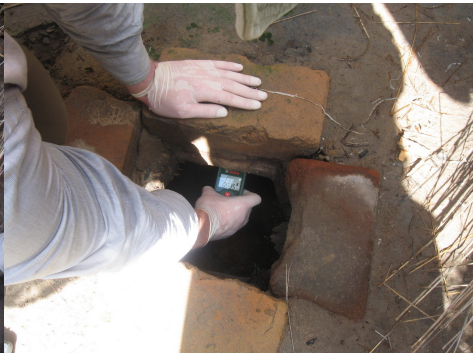

Supplement: SUPPLEMENTARY FIGURE S1 — (a) Example of pit latrine from Tanzania, (b) Sampling device to acquire liquid material from pit latrines, and (c) method for measuring fill-up rate. [file Image_1.PDF]

a)

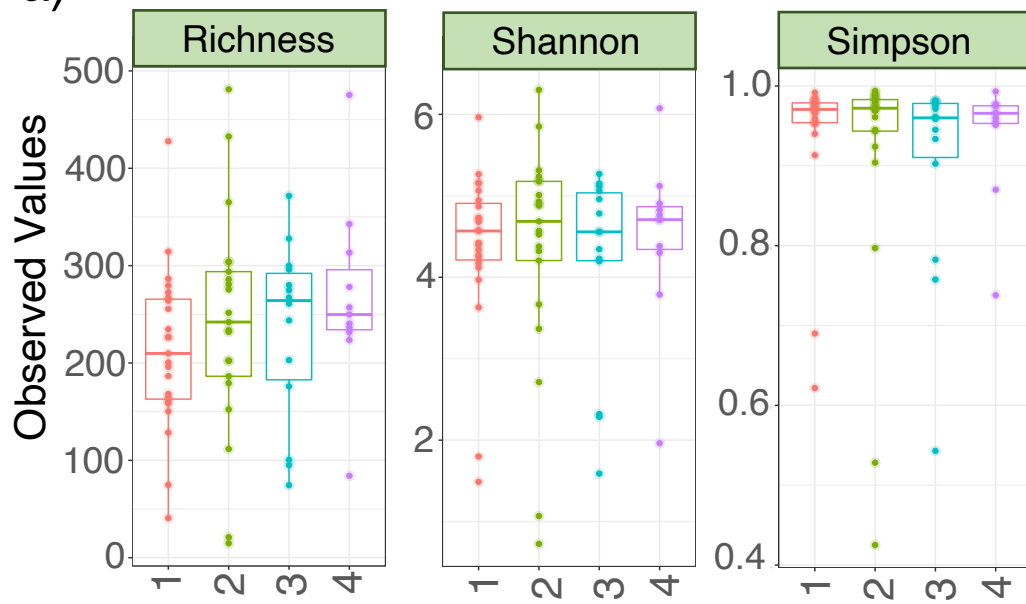

## Colour Legend

■ Depth 1 (20 cm)

■ Depth 2 (40 cm)

■ Depth 3 (60 cm)

■ Depth 4 (60 cm)

b)

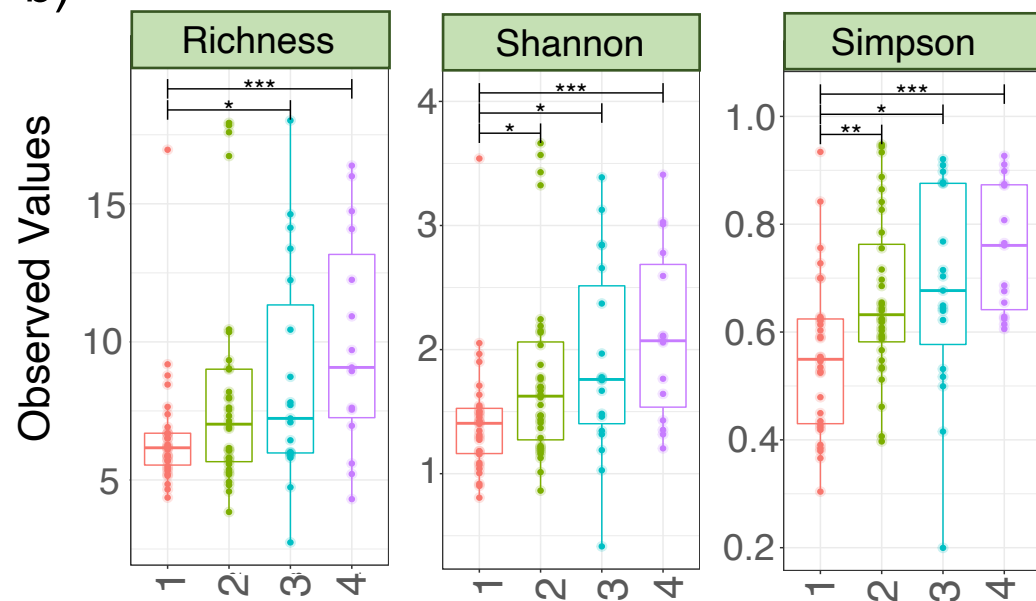

Supplement: SUPPLEMENTARY FIGURE S2 — (A) Alpha diversity metrics calculated on the rarefied microbiota data (OTUs at 3% divergence) from the four different pit latrine sample depths (Depth 1 = 20 cm, Depth 2 = 40 cm, Depth 3 = 60 cm, and Depth 4 = 80 cm); (B) Alpha diversity metrics calculated on the rarefied EnvO table returned from seqenv pipeline, indicating that the diversity of defined “descriptors” increased with increasing pit sample depth (Depth 1 = 20 cm, Depth 2 = 40 cm, Depth 3 = 60 cm, and Depth 4 = 80 cm). [file Image_2.PDF]

# Log-relative-normalised

## Environment-associated

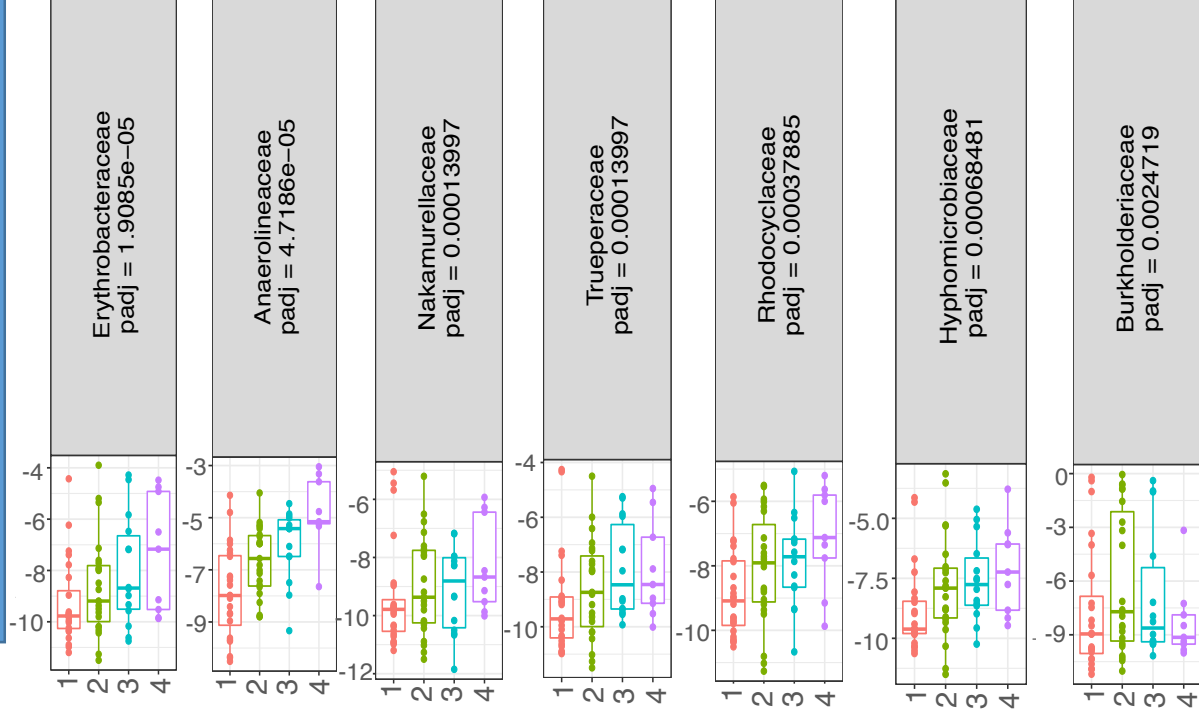

## Gut-associated

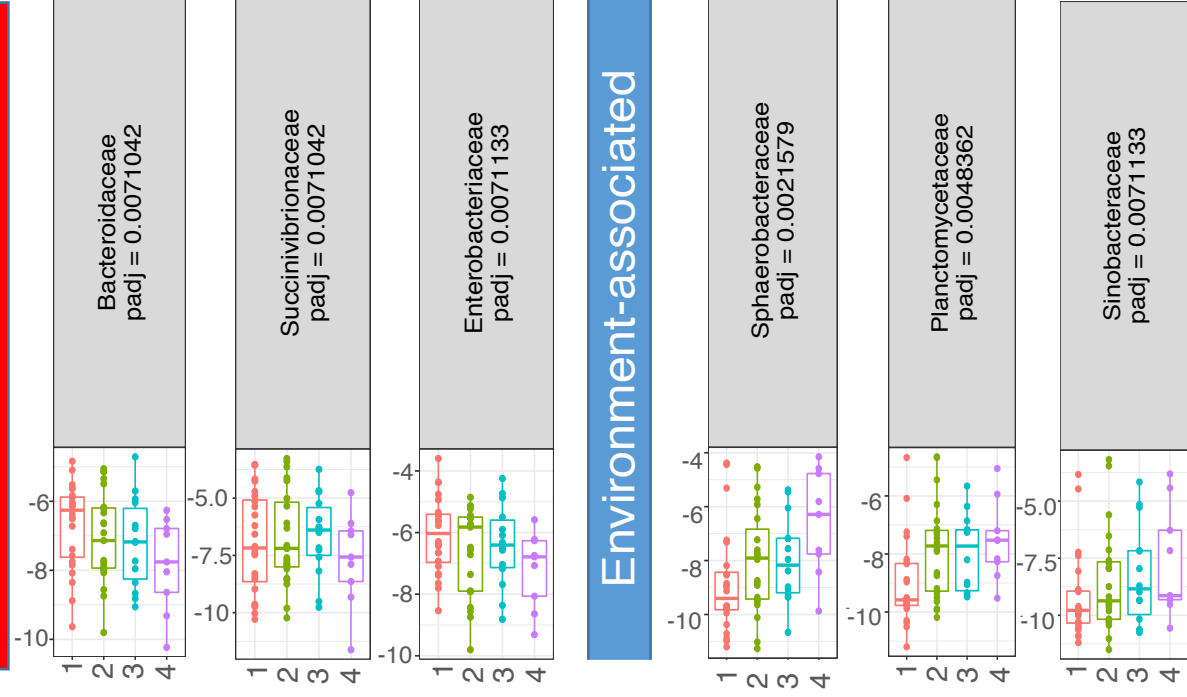

## Environment-associated

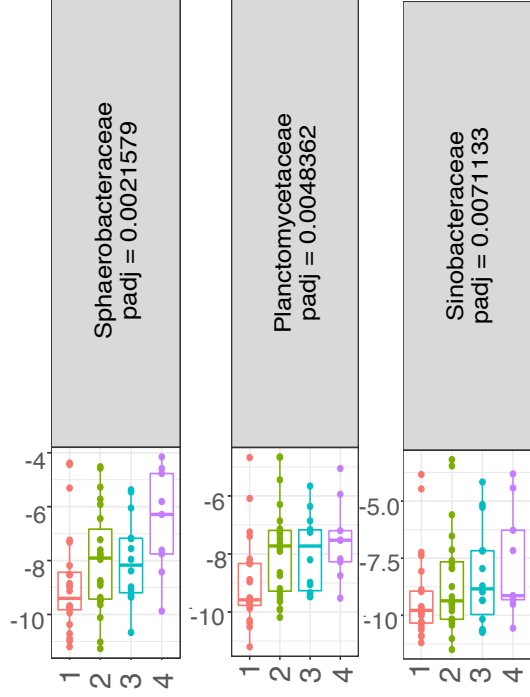

Supplement: SUPPLEMENTARY FIGURE S3 — Differentially abundant families not shown in Figure 4. [file Image_3.PDF]
