# Supplementary material for: Analysis of pit latrine microbiota reveals depth-related variation in composition, and key parameters and taxa associated with latrine fill-up rate
Source: Front Microbiol. 2022 Sep 23;13:960747. doi: 10.3389/fmicb.2022.960747 (PMC9539666; doi:10.3389/fmicb.2022.960747)
Supplement: SUPPLEMENTARY TABLE S1 — Characteristics of the 35 latrines from Tanzania used in the microbial analysis in this study. [file Table_1.DOCX]

**Supplementary Table 1:** Characteristics of the 35 study latrines.

|  | Tanzania  (Ifakara) |
| --- | --- |
|  |  |
| Latrine type (%) |  |
| Single Vault | 100 |
| Double Vault | 0 |
| Latrine ownership type (%) |  |
| Family | 78 |
| Shared | 16 |
| Communal | 6 |
| Nº Users (Mean (min-max)) | 8.5 (3-35) |
| Depth (Mean (min-max)) (m) | 1.45 (1.22-3.05) |
| Latrine structure (%) |  |
| Roof (Roof/No roof) | 83/17 |
| Wall (Grass-Bricks) | 49/51 |
| Vault (Lined-Unlined) | 26/74 |
| Slab type (%) |  |
| Soil/ Cement or brick | 64/36 |
| Soil type | Sandy/Loam |
| Climate |  |
| Rainfall average^&^ (min-max) (mm) | 120 (77-156) * |
| Temperature average (min-max) (°C) | 25 (19-32) * |
| Anal Cleansing | Water |
| Diet | Predominantly vegetarian |
| Excreta management | Disposal |
| Urine (%) |  |
| Disposed in pit/ Urine separators | 100/0 |

*Data from the last 10years

^&^Monthly average
